# Role of Chronic Alcoholism Causing Cancer in Omnivores and Vegetarians through Epigenetic Modifications

**DOI:** 10.1055/s-0040-1721814

**Published:** 2020-12-29

**Authors:** Syed Aaquil Hasan Syed Javid Hasan, Raisa Arifanie O'Zelian Pawirotaroeno, Syed Abrar Hasan Syed Javid Hasan, Elene Abzianidze

**Affiliations:** 1 Department of Acute Medicine, United Lincolnshire Hospitals NHS Trust, Lincoln, Lincolnshire, United Kingdom; 2 Department of Emergency, Mungra Medical Center, Nickerie, Suriname; 3 Department of Oncology, VS Hospital, Chennai, Tamil Nadu, India; 4 Department of Molecular and Medical Genetics, Tbilisi State Medical University, Tbilisi, Georgia

**Keywords:** chronic alcohol use, cancer epigenetic mechanisms, dietary deficiencies, epigenetic modifications, omnivores and vegetarians

## Abstract

One of the significant consequences of alcohol consumption is cancer formation via several contributing factors such as action of alcohol metabolites, vitamin deficiencies, and oxidative stress. All these factors have been shown to cause epigenetic modifications via DNA hypomethylation, thus forming a basis for cancer development. Several published reviews and studies were systematically reviewed. Omnivores and vegetarians differ in terms of nutritional intake and deficiencies. As folate deficiency was found to be common among the omnivores, chronic alcoholism could possibly cause damage and eventually cancer in an omnivorous individual via DNA hypomethylation due to folate deficiency. Furthermore, as niacin was found to be deficient among vegetarians, damage in vegetarian chronic alcoholics could be due to increased NADH/NAD ^+^ ratio, thus slowing alcohol metabolism in liver leading to increased alcohol and acetaldehyde which inhibit methyltransferase enzymes, eventually leading to DNA hypomethylation. Hence correcting the concerned deficiency and supplementation with S-adenosyl methionine could prove to be protective in chronic alcohol use.

## Introduction

One of the significant consequences of alcohol consumption is cancer formation. Approximately 3.6% of all cancer-related cases worldwide and 3.5% of all cancer-related deaths are attributed to chronic alcohol drinking. 1 Chronic alcohol consumption has been shown to cause epigenetic modifications thus forming a basis for cancer development. As omnivores and vegetarians differ in terms of nutritional intake and deficiencies, there could be different pathways through which chronic alcohol can induce damage in both these groups.

This article focuses on a brief account of the processes of alcohol-induced epigenetic modifications and the impact it could have on omnivores and vegetarians based on various published reviews, experimental trials, and randomized control trials. Finally, it highlights some of the more recent progress toward developing therapeutic agents in this promising target space.

## Methodology

The concept of epigenetics was understood by going through the Handbook of Epigenetics: The New Molecular and Medical Genetics, 2nd edition by Trygve Tollefsbol published by Elsevier. Several published reviews and studies were systematically reviewed from the following sources:

- Pubmed
- MedGen
- Taylor & Francis Online
- Elsevier

The search terms included “alcoholism and epigenetics,” “cancer and epigenetics,” “alcohol-induced hepatocellular carcinoma,” “vitamins and cancer,” and “nutrient deficiencies in omnivores and vegetarians,” “meat and cancer.” Reference lists of eligible papers were also searched.

## DNA Methylation

**Fig. 1:**
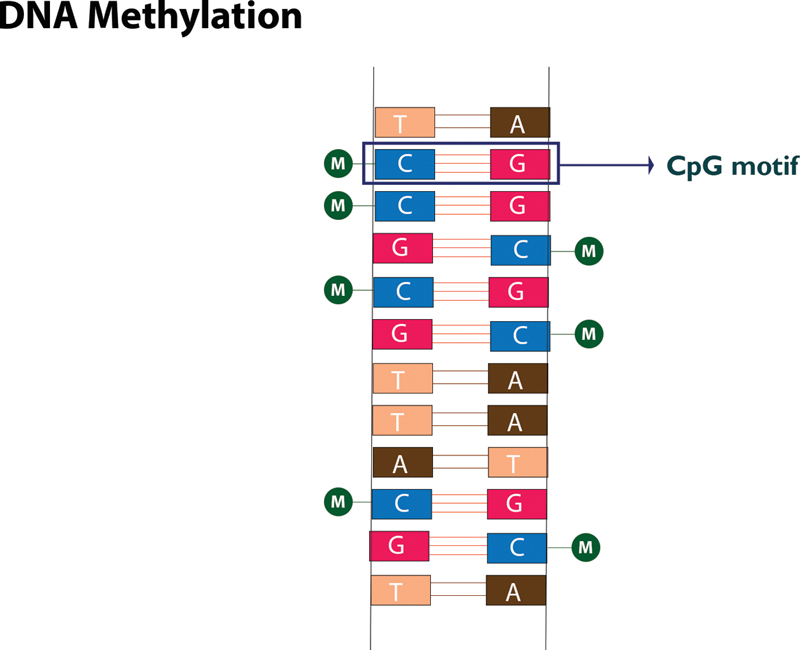
DNA methylation. Methylation at cytosine residues of CpG motifs silences genes. A, adenine; C, cytosine; G, guanine; M, methylated; T, thymine.

DNA methylation has been the center of discussion in several reviews. DNA methylation refers to donation of methyl group by S-adenosyl methionine (SAM) to a cytosine moiety of cytosine–guanine (CpG) dinucleotides in mammalian DNA catalyzed by family of enzymes called DNMTs 2 ( Fig. 1 ). CpG dinucleotide clusters to form CpG islands and methylation can take place in these islands and also in island shores (within 2kb from CpG island). Since CpG islands are located in approximately 60% of promoters, methylation in or near gene promoters results in gene silencing. 3 4 Hence DNA hypomethylation would result in gene activation.

**Fig. 2:**
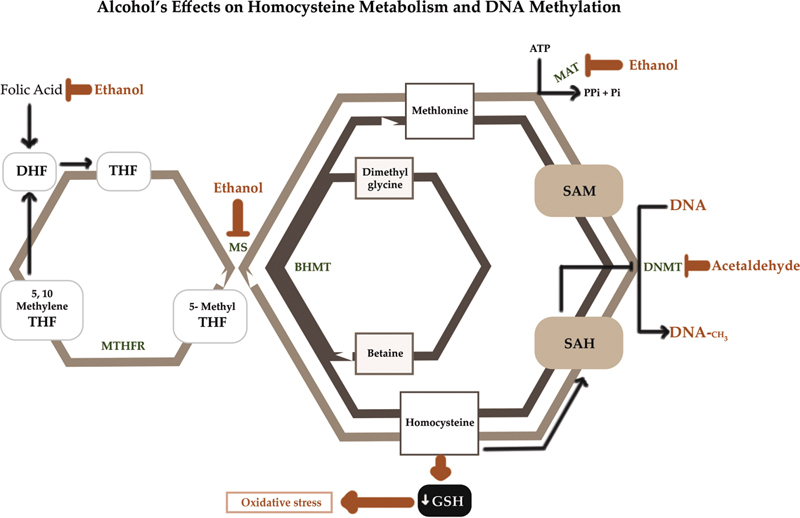
Alcohol's effects on homocysteine/methionine metabolism and DNA methylation. Methionine, which is formed by methylation of homocysteine (using either 5-methyltetrahydrofolate [5-methyl THF] or betaine as methyl donors), is essential for the production of S-adenosylmethionine (SAM), which in turn is used to methylate DNA. Chronic heavy drinking inhibits methionine synthase (MS), methionine adenosyltransferase (MAT), and DNA methyltransferase (DNMT) resulting in the reduction of methionine and SAM and the concurrent increase in homocysteine and S-adenosylhomocysteine (SAH). Increased SAH further inhibits DNA methyltransferases, ultimately resulting in global hypomethylation of DNA. ATP, adenosine triphosphate; BHMT, betaine homocysteine methyltransferase; GSH, glutathione; HCC, hepatocellular carcinoma; MAT, methionine adenosyltransferase; MTHFR, methylenetetrahydrofolate reductase; Pi, inorganic phosphate. (Reproduced with permission of Zakhari ^2^ ).

Once SAM donates a methyl group, it becomes S-adenosyl homocysteine (SAH), which then forms homocysteine by the action of SAH hydrolase. Homocysteine can either be remethylated to form methionine via a methyl group transfer either from N5-methyltetrahydrofolate (MTHF) by methionine synthase (MS), or from betaine by betaine homocysteine methyltransferase (BHMT) or enter transsulfuration pathway to form glutathione (GSH) 5 ( Fig. 2 ). SAH is a potent feedback inhibitor of DNMT and HMTs (homocysteine methyl transferases).

Ethanol interferes with DNA methylation in the following ways.

**Fig. 3:**
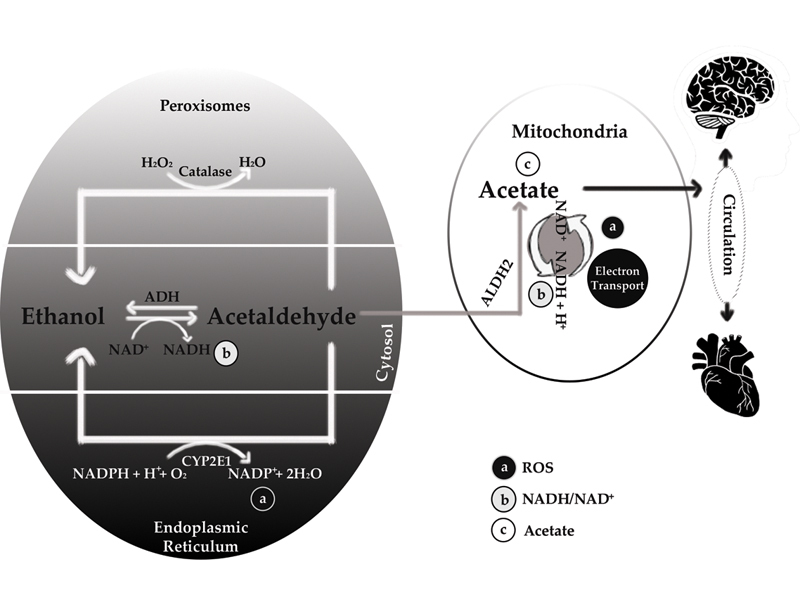
Oxidative pathways of alcohol metabolism. Alcohol is metabolized mainly in the cytosol by alcohol dehydrogenase (ADH) to produce acetaldehyde. At high levels of alcohol consumption, an enzyme in the endoplasmic reticulum, cytochrome P450 IIE1 (CYP2E1), becomes involved in metabolizing alcohol to acetaldehyde; this enzyme also is induced by chronic drinking. A catalase-mediated reaction in the peroxisomes is considered a minor metabolic pathway of alcohol metabolism. Acetaldehyde is further metabolized to acetate in the mitochondria. Alcohol metabolism results in the formation of NADH (reduced nicotinamide adenine dinucleotide) and thus changes the redox state of hepatocytes (i.e., increases the ratio of NADH/NAD ^+^ ). Both alcohol metabolism by CYP2E1 and the re-oxidation of NADH via the electron transport chain in the mitochondria results in the formation of reactive oxygen species (ROS). NAD ^+^ , nicotinamide adenine dinucleotide. (Reproduced with permission of Zakhari ^2^ ).

**Fig. 4:**
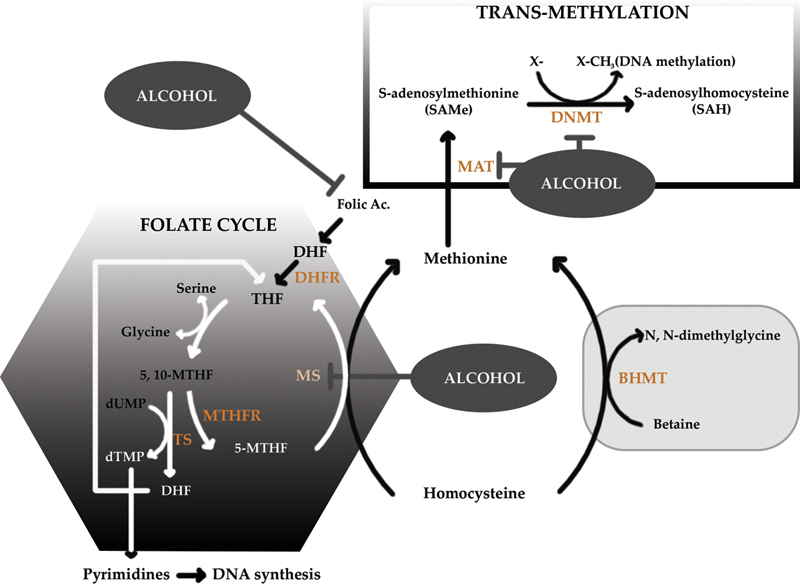
One-carbon metabolism with a schematic representation of the role of methionine in folate metabolism and transmethylation reactions and steps that are inhibited by alcohol. A methyl group is donated via folate metabolism by 5-methyltetrahydrofolate (5-MTHF) in the formation of homocysteine to methionine. Alcohol inhibits MS (methionine synthase) as well as reduces folate causing DNA hypomethylation, which could lead to tumor formation. Folate metabolism aids in DNA synthesis by donating another methyl group to deoxyuridine monophosphate (dUMP) to form deoxythymidine monophosphate (dTMP). Reduced folate levels cause an increased accumulation of dUMP leading to faulty DNA synthesis, subsequent DNA, and chromosomal damage and thus tumor formation. BHMT, betaine homocysteine methyltransferase; DHF, dihydrofolate; DHFR, dihydrofolate reductase; DNMT, DNA methyltransferase; Hcy, homocysteine; MAT, methionine adenosyl transferase; Met, methionine; 5-MTHF, 5-methyltetrahydrofolate; 5,10-MTHF, 5,10-methylenete-trahydrofolate; MTHFR, methylenetetrahydrofolate reductase; SAH, S-adenosylhomocysteine; SAM, S-adenosyl-methionine; THF, tetrahydrofolate; TS, thymidylate synthase. (Reproduced with permission of Varela-Rey et al ^4^ ).

- Transferase enzymes
- NAD ^+^ deficiency
- Reactive oxygen species (ROS) formation
- Folate deficiency

1. Transferase Enzymes
Ethanol inhibits methionine adenosyl transferase (MAT) and MS, thus inhibiting the formation of SAM 6 ( Fig. 2 ). BHMT is an alternative pathway that ensures conversion of homocysteine to methionine when MS is compromised by alcoholism ( Fig. 2 ). However, after extended periods of alcohol exposure this alternate pathway cannot be maintained. 4 Its methyl donor betaine is available in the diet and is also generated endogenously from choline, and betaine supplementation restores SAM levels in alcohol fed rats. 7
Decreased SAM/SAH ratio in the liver was noticed after ethanol feeding in rodent models of alcoholic liver disease (ALD) 8 and this decreased ratio results in feedback inhibition of DNMT ( Fig. 2 ).
Acetaldehyde, the first product of oxidative ethanol breakdown also affects DNMT activity in vitro 9 ( Fig. 2 ) and hence DNA methylation. Moreover, studies in rats treated with alcohol for 9 weeks 10 and in human patients with chronic alcoholism 11 showed reduced DNMT mRNA levels. Reduced DNMT activity leads to reduced SAM levels and decreased SAM/SAH ratio which in turn leads to a decrease in methylation of DNA.
2. NAD ^+^ /NADH
Ethanol oxidation pathway is also accompanied by the reduction of nicotinamide adenine dinucleotide (NAD ^+^ ) to NADH while forming acetaldehyde in cytoplasm and acetate in mitochondria by dehydrogenase enzymes ( Fig. 3 ). Hence alcohol metabolism increases NADH/NAD ^+^ ratio in the cytoplasm and mitochondria of hepatocytes 2 12 leading to alterations in hepatic lipid, carbohydrate, protein, lactate, and uric acid metabolism as well as modulation of gene expression. Increase in NADH results in inhibition of sirtuin1 (SIRT1), thereby causing histone acetylation resulting in gene activation. 2
As ethanol oxidation pathway depletes NAD ^+^ leading to increased NADH/NAD ^+^ ratio, alcohol metabolism decreases slowly overtime leading to increased acetaldehyde and alcohol in the liver leading to inhibitions of methyl transferase activities as briefed above, paving way for DNA hypomethylation.
3. ROS formation
During oxidation reaction of alcohol by cytochrome P450 2E1 (CYP2E1), reactive oxygen species (ROS) are also generated 13 ( Fig. 3 ). A study showed that when genetically modified cultured liver cells producing excessive CYP2E1 were incubated with ethanol, DNA damage by ROS occurred owing to the formation of 8-hydroxy-2'-deoxyguanosine (8-OHdG), 13 an abnormal variant of a nucleotide that can result in a decrease in methylated DNA during DNA repair. 14 8-OHdG can be incorporated into CpG islands which then inhibit the methylation of adjacent cytosine residues by enzymes called methyl transferases, resulting in hypomethylation. 15
Another way by which excessive ROS formation can decrease DNA methylation is by acutely depleting GSH and thus diverting homocysteine to transsulfuration pathway to form GSH, rather than producing methionine and SAM 2 ( Fig. 2 ).
4. Folate
Dietary folate contributes to the production of 5-methyltetrahydrofolate (5-MTHF) in the DNA synthesis pathway. 5-MTHF is the initial methyl donor to the substrate homocysteine thus forming methionine by the action of Vit B12 dependent MS which in turn forms SAM with the help of MAT and ATP 16 ( Fig. 4 ).
  Several studies over the years have shown folate deficiency in chronic alcoholism due to several causes.

- Folate absorption: In chronic alcoholics coming to hospital emergency rooms for alcohol withdrawal, two studies showed a decrease in absorption of 3H-labeled folic acid in comparison to acute ingestion of alcohol. 17 18
An in vitro study 19 in chronic ethanol fed micropigs demonstrated decreased expression of reduced folate carrier (RFC) by isolated jejunal brush border membranes, and a recent study in chronic ethanol fed rats also demonstrated decreased intestinal mucosal expressions of RFC and proton coupled folate transport (PCFT). 20 Both RFC and PCFT are necessary in folate absorption and transport.
- Folate storage: A study involving alcohol fed rats noticed decreased folate storage enzyme in the liver called folylpolyglutamate synthetase. 21
- Folate urinary excretion: Loss of folate in the urine has been documented in chronic alcoholic subjects, 22 ethanol fed rats, 23 and in chronic ethanol fed monkeys. 24
Hence folate deficiency in chronic alcoholics can contribute to low levels of SAM and thus a decrease in SAM/SAH ratio 25 leading to DNA hypomethylation.

The net effect of all these alcohol-induced reduction in methyltransferase activity, reduced synthesis of SAM, increased NADH/NAD ^+^ ratio, ROS-induced DNA damage and folate deficiency is reduced SAM/SAH ratio, leading to DNA hypomethylation.

## Carcinogenesis

Based on epidemiological data, it has been concluded that carcinogenesis in numerous organs, including the upper aerodigestive tract, liver, colorectum, and female breast is induced by alcohol. 26 Chronic alcohol use of greater than 80 g/d for more than 10 years increases the risk for hepatocellular carcinoma (HCC) approximately fivefold. Furthermore, the risk for HCC in decompensated alcohol-induced cirrhosis approaches 1% per year. 27 Interestingly, HCC is more likely to develop 1 to 10 years after the cessation of drinking. 28

A study reported explains the changes in gene expression and liver injury through epigenetic modifications induced by chronic ethanol feeding and not by acute feeding. The results support the concept that chronic ethanol ingestion induces altered gene expression as a result of changes in epigenetic mechanisms, where acetylation and methylation of histones were altered. 29

### • DNA Methylation

All the above-mentioned pathways lead to global DNA hypomethylation. HCC with several etiologies, including chronic alcoholism, is associated with a significant global DNA hypomethylation, which may result in malignant transformation through mechanisms such as loss of imprinting and chromosomal instability. 30 31 Altered methionine metabolism and the subsequent hypomethylation is one mechanism by which alcohol produces ALD and HCC. 32

### • Histone Acetylation

**Fig. 5:**
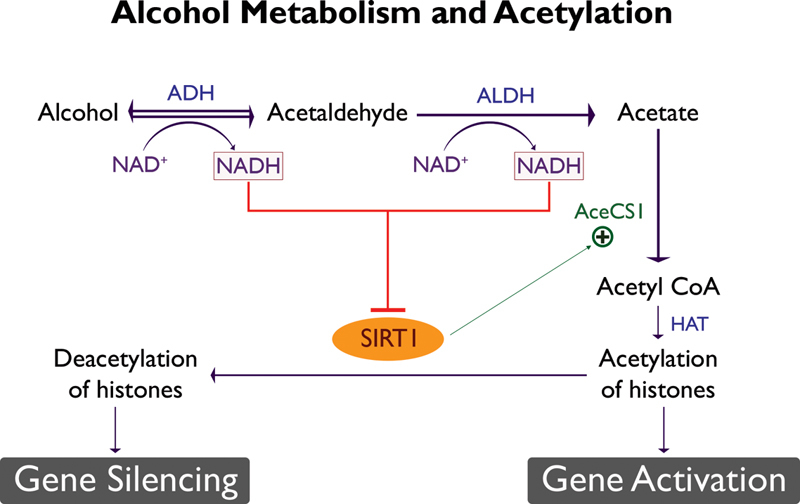
Interaction between alcohol metabolism and acetylation. Sirtuin1 (SIRT1) while being a deacetylase enzyme, acts as a regulator of gene transcription by activating Acetyl CoA Synthase1 (AceCS1). In chronic alcoholism, oxidative alcohol metabolism results in increased NADH/NAD ratio which inhibits SIRT1 and hence leads to increased histone acetylation via histone acetyltransferase and gene activation. ADH, alcohol dehydrogenase; ALDH, aldehyde dehydrogenase; NAD ^+^ , nicotinamide adenine dinucleotide; NADH, reduced nicotinamide adenine dinucleotide. (Reproduced with permission of Zakhari ^2^ ).

**Fig. 6:**
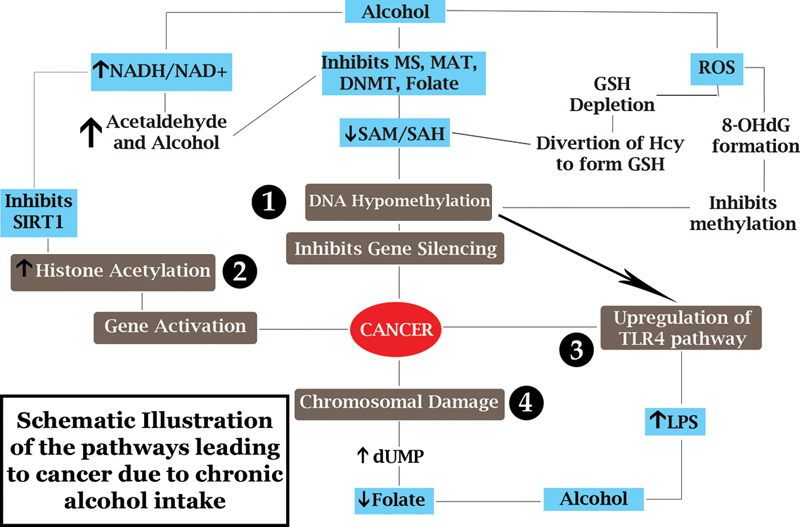
Schematic illustration of the pathways leads to cancer due to chronic alcohol intake. Alcohol inhibits various enzymes and folate as described, alters the ratios of SAM/SAH and NADH/NAD ^+^ , increases reactive oxygen species (ROS) formation and lipopolysaccharides (LPS), all of these leading to cancer via: (1) DNA hypomethylation. (2) Increased histone acetylation. (3) Upregulation of toll-like receptor 4 (TLR4) pathway. (4) Chromosomal damage. DNMT, DNA methyltransferase; dUMP, deoxyuridine monophosphate; GSH, glutathione; Hcy, homocysteine; 8OHdG, 8-hydroxy-2'-deoxyguanosine; MS, methionine synthase; MAT, methionine adenosyl transferase; NAD ^+^ , nicotinamide adenine dinucleotide; NADH, reduced nicotinamide adenine dinucleotide; SAH, S-adenosylhomocysteine; SAM, S-adenosyl-methionine; SIRT1, Sirtuin 1.

Chronic ethanol feeding in rats alters the methylation and acetylation of histones in the liver. 29 33 Histone acetylation globally activates gene expression 29 as well as increases the levels of a deacetylase (i.e., SIRT1) in alcohol-fed animals. 29 33 SIRT1 activates mammalian acetyl-CoA synthase, resulting in the formation of acetyl-CoA which is then used to acetylate histones, resulting in gene activation. Subsequently, SIRT1 deacetylates the histones, resulting in gene silencing. Thus, SIRT1 can act as a sensor to regulate gene transcription. 2 Furthermore, increased NADH resulting from oxidative alcohol metabolism inhibits SIRT1, thereby affecting deacetylation ( Fig. 5 ) thus promoting gene activation ( Fig. 6 ).

### • Toll-Like Receptor Pathway

Another study shows that the transformation to HCC is driven by the toll-like receptor 4 (TLR4) signaling pathway, which is upregulated by combined effect of an increase in lipopolysaccharides levels in the liver that result from alcohol abuse and at epigenetic level by inhibition of DNA methylation 34 ( Fig. 6 ). This upregulation of the TLR4 pathway, which has been demonstrated in rats chronically fed ethanol, can be prevented by SAM supplementation. 35 36

### • Chromosomal Damage

Increased DNA instability and aberrant DNA methylation patterns are the two main mechanisms described that may explain the cancer-promoting effects of limited folate levels. Folate deficiency alters the balance of the pool of nucleotides needed for the synthesis of new DNA molecules, leading to dUMP accumulation ( Fig. 6 ). As a result, dUMP is misincorporated into new DNA molecules; this and the subsequent repair processes can lead to double-strand breaks in the DNA and chromosomal damage, ultimately resulting in cancer. 4 The aberrant DNA methylation patterns associated with folate deficiency are the result of folate's role in one-carbon metabolism as described above ( Fig. 4 ).

## Omnivores versus Vegetarians

A study in Switzerland demonstrated that the highest prevalence for vitamin and mineral deficiencies was as follows: in the omnivorous group, for folic acid (58%); in the vegetarian group, for vitamin B6 and niacin (58 and 34%, respectively). 37 Another survey showed that folate intake was lower for omnivores while niacin was lower for vegetarians. 38

Studies have shown that low folate intake coupled with high alcohol intake increases the risk for colon cancer 39 40 41 and heavy alcohol consumption increases the risk of breast cancer to 40 to 50%. 42 43 A review has described the incidence of folate deficiency in chronic alcoholism and documented the evidence of its involvement in hepatic methionine metabolism, DNA hypomethylation, pathogenesis of ALD and thus an increased risk for hepatocellular cancers. 32

Alternatively, prospective cohort study in Australia, Sweden including a cohort from 10 European countries concluded that the consumption of fresh red meat and processed meat seemed to be associated with an increased risk of colorectal cancer. 44 45 46 A meta-analysis of colorectal cancer in 10 cohort studies reported a statistically significant dose–response relationship, with a 17% increased risk (95% CI 1.05–1.31) per 100 g/d of red meat and an 18% increase (95% CI 1.10–1.28) per 50 g/d of processed meat. 47 Cohort studies and population-based case control studies have shown positive associations between consumption of red meat and cancers of the pancreas and the prostate (mainly advanced prostate cancer), and between consumption of processed meat and cancer of the stomach. 48

As described above, red or processed meat consumers with folate deficiency may lead to several types of cancers, especially colorectal cancer at a faster rate.

On the other hand, the vegetarians show niacin deficiency. The dietary status of niacin (vitamin B3) has the potential to influence DNA repair, genomic stability, and the immune system, eventually having an impact on cancer risk. Niacin, in the form of NAD, participates in a wide variety of ADP-ribosylation reactions. 49 Studies in rats suggest that inhibition of NAD ^+^ ADP ribosyltransferase and associated DNA repair play an important role in the early initiating stage of liver carcinogenesis. 50 Another study demonstrates that reduced NAD ^+^ concentrations in humans lead to HCC. 51

## Therapeutic Options

Various studies have suggested that folate, niacin, betaine, and SAM could prevent ALD progression and tumor formation.

Christensen et al 52 showed a strong trend toward decreased DNA methylation with increasing alcohol intake, and a trend toward increased methylation with increasing dietary folate, thus suggesting folate intake could prevent cancer development.

A study using rat hepatoma cells (cultured with ROS generating system) with increased invasive capacity demonstrated that niacin and trigonelline suppressed this ROS-potentiated invasive capacity. 53 Furthermore, supplementation of NAD ^+^ pools via dietary nicotinamide riboside in high cancer risk mice not only reduced dysplastic lesions, but also prevented tumor development. 51

In the folate-deficient ethanol fed micropig model, experimental SAM treatment was shown to prevent ALD and its gene expressions for lipogenesis, apoptosis, and oxidative liver injury. 54 55 Contrastingly, in a double blinded randomized controlled trial, it was concluded that SAM was no more effective than placebo for the treatment of ALD with the limitation of relatively small numbers of patients. 56

Several studies in animal models suggested the possible mechanisms of action of betaine which include increased SAM hepatic levels, reduced SAH levels by increased homocysteine metabolism thus improving SAM/SAH ratio, 57 increased metabolic rate and generation of NAD ^+^ for alcohol oxidative metabolism by alcohol dehydrogenase, 58 inhibition of TLR-4 in the inflammation pathway, 59 and protection against apoptosis. 60

To summarize, dietary folate and niacin could be protective in cancer development. While there is abundant evidence that SAM and betaine are protective in the development of experimental ALD in animal models, the efficacy of SAM as a treatment modality of established ALD has not been proven conclusively in clinical trials. 32

## Conclusion

DNA hypomethylation in chronic alcoholism has been shown to be at the center of cancer development. It can be caused by several ways, especially folate and niacin deficiencies. With studies showing a reduced folate intake as well as reduced plasma levels in omnivores, chronic alcoholism could potentiate the process of colorectal cancer and possibly HCC. Subsequently niacin deficiency in chronic alcoholic vegetarians could result in an increased risk of tumor formation as well. Further studies are required to link cancer development in chronic alcoholics based on contrasting diets and to prove this clinically. If proven, this could very well help in therapeutic applications in the form of folate supplementation in omnivores and niacin supplementation in vegetarians to prevent cancer development or ALD progression. Upregulation of the TLR4 pathway which has been shown to drive transformation of progenitor cells to HCC could be prevented by SAM supplementation as shown in chronically fed rat models. The protective role of SAM and betaine still remains unclear in clinical trials and further studies are necessary to confirm this.
